# Stability of Flavan-3-ols, Theaflavins, and Methylxanthines in 30 Industrial Green, Black, and White Tea (*Camellia sinensis* L.) Extracts Characterized via Liquid Chromatography Techniques

**DOI:** 10.3390/antiox12122121

**Published:** 2023-12-15

**Authors:** Antonio M. Inarejos-Garcia, Julia Heil, Sonia Guilera Bermell, Gertrud E. Morlock

**Affiliations:** 1 Department of Functional Extracts, ADM® Wild Valencia, 46740 Carcaixent, Spain; 2 Institute of Nutritional Science, Chair of Food Science and TransMIT Center for Effect-Directed Analysis, Justus Liebig University Giessen, Heinrich-Buff-Ring 26-32, 35392 Giessen, Germany; 3 Center for Sustainable Food Systems, Justus Liebig University Giessen, Senckenbergstr. 3, 35390 Giessen, Germany

**Keywords:** oxidative stability, catechins, polyphenols, antioxidants, bioactive molecules, commercial tea extract, purified tea extract, liquid chromatographic techniques

## Abstract

Commercially available tea extracts for dietary supplements and nutraceuticals are standardized to characteristic components of *Camellia sinensis* L., such as epigallocatechin gallate (EGCG) and total catechins or polyphenols. However, since most commercial tea extracts are highly concentrated into only one molecule such as EGCG, the comparatively less stable catechin, the oxidative stability of the extract during the 24-month shelf life was questioned. It was hypothesized that the overall oxidative stability is reduced for highly purified/concentrated tea extracts due to the absence of other natural antioxidants stabilizing the complex mixture. Via liquid chromatographic analysis, the individual chromatographic profiles of 30 commercial white, green, and black tea extracts were evaluated and compared regarding oxidative stability and functional properties. The contents of bioactive flavan-3-ols, theaflavins, and methylxanthines differed much from what was claimed by the suppliers. At the end of the product shelf life, most of the commercial green and black tea extracts showed a decrease in the flavan-3-ol content, the main bioactive components of tea. A high EGCG content to the detriment of other possibly stabilizing flavan-3-ols or antioxidants in tea was found to explain the lower oxidative stability of such tea extract products. A natural overall composition of molecular structures was found to be superior to a strong enrichment in just one molecule.

## 1. Introduction

Tea is produced by drying and stabilizing leaves from *Camellia sinensis* L. [1]. Green and white tea leaves have significantly higher contents of monomeric flavan-3-ols, such as (+)-catechin (C), (−)-epicatechin (EC), (−)-epicatechin gallate (ECG), (−)-epigallocatechin (EGC), (−)-gallocatechin gallate (GCG), (−)-epigallocatechin gallate (EGCG), and (+)-gallocatechin (GC), compared to oxidated tea leaves such as black tea [2,3]. Black tea presents a significant content of oligomeric and polymeric forms from flavan-3-ols, chiefly theaflavins and thearubigins. The traditional crushing of tea leaves releases the enzyme polyphenoloxidase, which catalyzes the polymerization of monomeric flavanols that also contribute to the organoleptic properties of the product apart from being bioactive components [4].

Catechins are flavan-3-ols, naturally present in tea, with significant antioxidant capacity to scavenge different reactive oxygen radicals by virtue of the reducing properties of the multiple hydroxyl groups attached to the aromatic rings (phenolic groups) [5]. Methylxanthines, such as caffeine, theophylline, and theobromine, are another important group of bioactive components for green and black tea leaves [6]. Apart from the initial composition of the raw material, the concentration of these compounds in the finished extract depends on the processing technology [7]. Henning et al. [3] studied the antioxidant capacity of individual components from *Camellia sinensis* using the ORAC methodology, comparing them to ascorbic acid as the reference antioxidant, and showed that gallic acid and monomeric catechins (EGC, EC, C, ECG, GCG, and EGCG) exhibited even higher antioxidant capacity than ascorbic acid. The functional properties of tea are well-known, and the consumption of tea increases every year not only as a beverage, but also as functional extracts included into different food and dietary supplement products, some of them related to health and wellness. Functional tea extracts in the market may be found under different specifications, standardizations, qualities, *etc*., characterized by different analytical methods to quantify the bioactive molecules. In addition, the most dietary supplements based on tea extracts are usually formulated together with vitamins and/or minerals in a concrete concentration [7], just to claim different health properties (antioxidant, weight loss, cardiovascular, detox, immune system, mood, *etc*.). For example, in Europe, this is regulated according to “general function” claims under Article 13.1 of the EC Regulation on Nutrition and Health Claims [8].

High-performance liquid chromatography (HPLC) and high-performance thin-layer chromatography (HPTLC) are the most common analytical techniques used for the profiling, identification, and quantification of bioactive compounds in tea [9,10,11]. Unfortunately, there is no agreed harmonized reference method due to the natural complexity of tea and its extracts. New and faster methods for a more complete and precise quantification of tea components as well as characteristic molecules for the detection of adulteration and fraud are currently being targeted. Recently, a new regulation on EGCG dosing has been published in Europe, setting a maximum dose of 800 mg/day for safety reasons [12], which could affect highly purified tea extracts standardized to EGCG. In terms of oxidative stability, catechins, especially EGCG, are susceptible to degradation throughout the storage period. Previous research has investigated the shelf life of tea leaves in their original containers and even of ready-to-drink tea beverages, controlling the stability of catechins and other tea components at room temperature [13,14]. In addition to tea leaves and beverages, hundreds of dietary supplements based on tea extracts have become available on the market in recent years, but to our knowledge, the oxidative stability of the entire profile of functional molecules contained in these extracts has not been studied.

In this research study, the oxidative stability of 30 different commercial green, black, and white tea extracts during the 24-month shelf life was questioned and thus investigated. Therefore, their flavan-3-ol, theaflavin, and methylxanthine contents had to be studied using HPLC–diode array detection (DAD) and HPTLC–UV/Vis (using a simple derivatization with the Fast Blue B salt reagent). Unknown components should further be characterized via HPLC–electrospray ionization-triple quadrupole time-of-flight mass spectrometry (ESI–QTOF-MS/MS).

## 2. Materials and Methods

### 2.1. Reagents and Chemicals

The three methylxanthine standards, caffeine (Caf, 100%), theobromine (TB, 98%), and theophylline (TP, 99.5%), gallic acid (GA, 100%), and theaflavin (98.9%) were purchased from Phytolab, Vestenbergsgreuth, Germany. The seven monomeric flavan-3-ol standards sold as green tea catechin mix, composed of (+)-catechin (C, 98.5%), (−)-epicatechin (EC, 98.9%), (−)-epicatechin gallate (ECG, 98.5%), (−)-epigallocatechin (EGC, 99.5%), (−)-gallocatechin gallate (GCG, 99.6%), (−)-epigallocatechin gallate (EGCG, 97.9%), and (+)-gallocatechin (GC, 99.6%), (−)-catechin 3-gallate (CG, 99.5%), and the theaflavin mix (tea extract from *Camellia sinensis*, 99.5%) were obtained from Merck, Madrid, Spain. Fast Blue B salt (95%) was delivered by MP Biomedicals, Eschwege, Germany. HPTLC plate silica gel 60 RP18 WF_254s_, trifluoroacetic acid, acetonitrile, methanol, dimethyl sulfoxide, citric acid monohydrate and water, all of chromatographic quality, were purchased from VWR, Llinars del Vallés, Spain, or Darmstadt, Germany. Bidistilled water used for HPTLC was generated by Heraeus Destamat Bi–18E, Thermo Fisher Scientific, Dreieich, Germany.

### 2.2. Commercial Tea Extracts

A total of 26 different powdered tea extracts were bought as dietary supplements or nutraceuticals on the market, whereas 4 tea extracts were self-produced (Table 1). A total of 30 powdered extracts of tea (*Camellia sinensis* L.) claimed 100% water extraction in the corresponding technical documentation. Among the 19 commercial green tea extracts, 6 (C1, C2, C8, C9, C19, and C20) were standardized to polyphenols, catechins and EGCG, 6 (C5, C6, C15–C18) to polyphenols/catechins, 3 (C7, C13, and C14) to EGCG only, and 4 (C3, C4, C21, and C22) to L-Theanine, a non-proteinogenic amino acid of *Camellia sinensis*. The seven black tea extracts (C10–C12 and C23–C26) were also standardized to five different compound groups and its combinations.

### 2.3. Storage Conditions

The commercial samples (Table 1), once arrived at the laboratory, were divided into replicates and stored according to the supplier recommendation at room temperature, isolated from humidity and light in aluminum bags. Thus, all commercial extracts were quantified twice via HPLC–DAD, *i.e*., in the beginning (initial analysis) and after 23 months of storage, just before the end of the stated 24-month shelf life.

### 2.4. Sample Preparation and Standard Solutions

For the HPLC quantification, each powdered tea extract (Table 1, 0.1 g each) was dissolved in 10 mL dimethyl sulfoxide (methanol for method comparison with HPTLC) in a 10-mL centrifuge tube (10 mg/mL), followed by shaking at maximum shaking velocity for 30 min (orbital shaker model Grant bio PSU-10i, VWR) and filtration through a 0.45-µm syringe nylon filter prior to analysis. Four calibration standard solutions (EGCG, caffeine, theaflavin, and gallic acid) were prepared as 1000, 500, 200, 100, and 10 mg/L in dimethyl sulfoxide. For HPTLC analysis, samples were dissolved in methanol (10 mg/mL) and further 1:4 diluted with methanol (400 µL extract plus 1200 µL methanol, 2.5 mg/mL). Only one methanolic calibration standard mixture solution of EGCG, theaflavin, and caffeine (37 µg/mL each) was needed and prepared. All solutions were stored at −20 °C in the dark.

### 2.5. Quantification via HPLC–DAD Analysis

The HPLC equipment consisted of a Shimadzu Nexera XR UHPLC 70 MPa coupled to a DAD SPD-M40 model (Izasa Scientific, Madrid, Spain). The chromatographic separation of flavan-3-ols, methylxanthines, theaflavins, and gallic acid [15] was performed using an octadecyl silane column Zorbax Eclipse Plus C18 (250 mm, 4.6 mm, 5 µm) including C18 precolumn (12.5 mm, 4.6 mm, 5 µm) from Agilent Technologies, Madrid, Spain, with the column oven temperature set to 32 °C. The injection volume was 2 µL. The binary gradient system consisted of 5% acetonitrile plus 0.035% trifluoroacetic acid as phase A, and 50% acetonitrile plus 0.025% trifluoroacetic acid as phase B, all *v*/*v* in water. The 30 min gradient started with A-B 90:10 at a flow rate of 1.0 mL/min, increased to 20% B at 10 min, 40% B at 16 min, 50% at 20 min, and decreased to 40% B from 25 to 27 min. The column was re-equilibrated to the initial conditions during 3 min before injection. Detection (absorbance measurement) was performed at 275 nm for methylxanthines, catechins, theaflavins, and gallic acid. The sum of monomeric flavan-3-ols was quantified as EGCG equivalents, that of methylxanthines as caffeine equivalents, that of theaflavins as theaflavin equivalents, and gallic acid was analyzed individually. External calibration curves were performed with at least 5 different concentration points (r^2^ ≥ 0.99), with individual formulas such as EGCG (y = 3613 x + 103,316), caffeine (y = 6334 x + 19,537), theaflavin (y = 4548 x + 2394), and gallic acid (y = 6916 x − 6308). Samples were analyzed twice (*n* = 2).

### 2.6. Theaflavin Extraction from Black Tea Leaves

In order to evaluate the most appropriate conditions to extract theaflavins from black tea leaves, a parallel experiment was performed with 100% ethanol, 100% water, and different volume proportions of ethanol/water mixtures (1:1, 3:7, and 7:3) at laboratory scale. Briefly, 100 g black tea leaves (reference raw material) was extracted with 1 L each of the mentioned extractants at 70 °C (below the ethanol boiling point) in a heated bath coupled to an overhead stirrer (OS20, Labbox Labware, Premia de Dalt, Spain) for 30 min. The resultant extract was concentrated (Hei-VAP Expert Control Rotary evaporator, Heidolph, Schwabach, Germany), and then reduced to powder in a vacuum oven (Vaciotem-T, Selecta, Barcelona, Spain), and finally dissolved in dimethyl sulfoxide for the HPLC analysis of theaflavins as described.

### 2.7. Identification via HPLC–ESI–QTOF-MS/MS Analysis

Additional unknown signals detected in the four ADM^®^ tea extracts were identified via HPLC–ESI–QTOF-MS/MS. The separation was performed analogously; however, the flow rate was set to 0.6 mL/min and the Triple TOF 5600 MS/MS (QTOF) detector with software PeakView 2.2 (AB SCIEX, Atlanta, GA, USA) was used. Full-scan ESI-MS spectra (*m*/*z* 80–1300) in the negative ion mode were recorded using capillary voltage 3.0 kV, extractor voltage 4.0 V, cone voltage 30 V, ion source temperature 150 °C, desolvation temperature 300 °C, and desolvation gas flow 600 L/h.

### 2.8. Quantification via HPTLC-UV/Vis Analysis

The solution of the powdered ADM^®^ Tea Complex extract (2 µL/band each, 2.5 mg/mL) and increasing volumes of the standard mixture solution of EGCG, theaflavin, and caffeine (S1–S5: 1, 1.5, 4.5, 10.5, and 22.5 µL/band, 37 µg/mL) were applied (Automatic TLC Sampler 4, CAMAG, Muttenz, Switzerland) on the HPTLC plate silica gel 60 RP18 WF_254s_ (prewashed with methanol water 4:1). The development was performed using 3 mL of a mixture of 1.2 mL acetonitrile, 4 mL water, and 15 mg citric acid (Twin-Trough Chamber 10 cm × 10 cm, CAMAG), taking 30 min [11]. The relative humidity of the surrounding air was 35%. The documentation of the chromatogram was performed at 254 nm (UV), 366 nm (FLD), and under white light illumination (Vis; TLC Visualizer, CAMAG). The chromatogram was detected via absorbance measurement (TLC Scanner 4, CAMAG) at 275 nm using the deuterium lamp (methylxanthines and theaflavins) and at 546 nm using the mercury lamp after derivatization with 0.5% aqueous Fast Blue B salt reagent (flavan-3-ols), applied homogeneously via piezoelectric spraying using the red nozzle at spraying level 6 (Derivatizer, CAMAG), followed by plate heating at 100 °C for 3 min (TLC Plate Heater, CAMAG).

## 3. Results and Discussion

### 3.1. Outline and Focus of the Study

**Figure 1:**
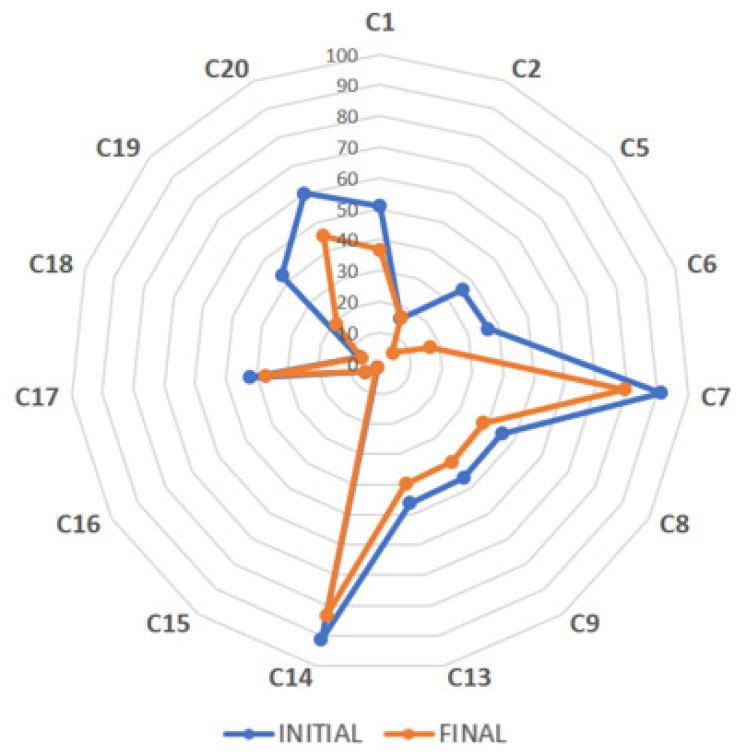
Spider map showing the EGCG content (%, dry basis) of 15 commercial green tea extracts (C1–C20) analyzed via HPLC–DAD initially and finally at the end of the product shelf life (23 months later).

**Figure 2:**
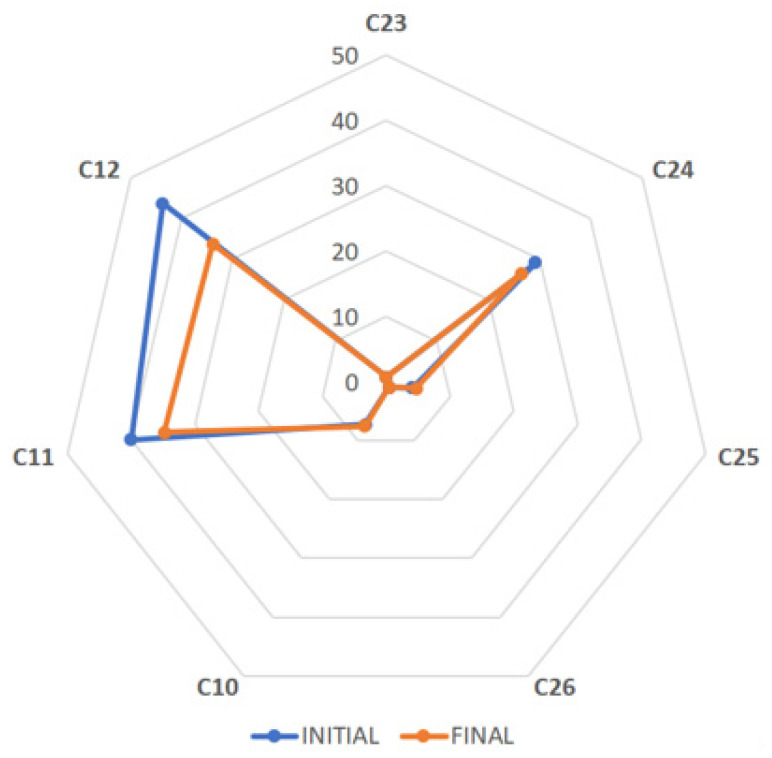
Spider map showing the EGCG content (%, dry basis) of seven commercial black tea extracts (C10–C26), all analyzed via HPLC–DAD initially and finally at the end of the product shelf life.

**Figure 3:**
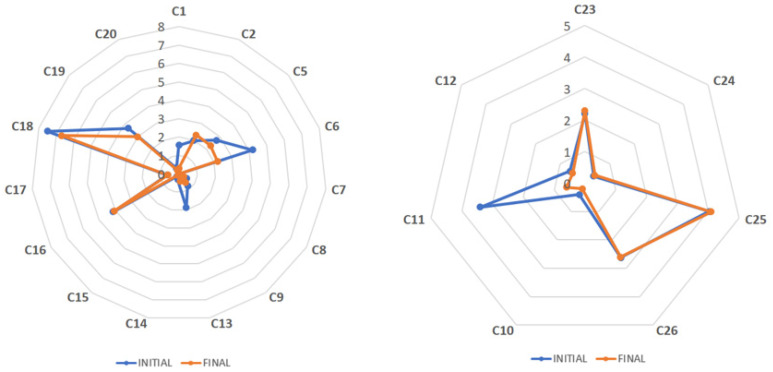
Spider maps showing the caffeine content (%, dry basis) of 15 commercial green and seven black tea extracts, all analyzed via HPLC–DAD initially and finally at the end of the product shelf life.

**Figure 4:**
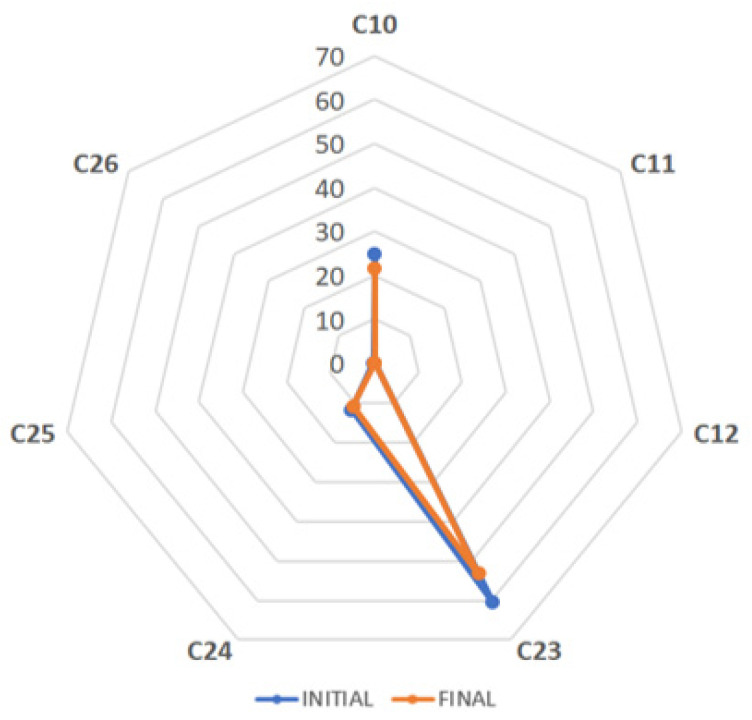
Spider map showing the content of theaflavins (%, dry basis) of seven commercial black tea extracts, all analyzed via HPLC–DAD initially and finally at the end of the product shelf life.

After samples (Table 1) arrived at the laboratory, important bioactive compounds were quantitatively analyzed within two weeks (initial analysis) using an internally validated HPLC–DAD method, established according to [15]. The individual EGCG contents of 15 green and 7 black tea extracts were obtained, presented as spider maps (Figure 1 and Figure 2, respectively). The four green tea extracts standardized to L-theanine were excluded because their obtained chromatographic profiles differed (Figures S1–S4) due to the different standardization compared to the other tea extracts, which were chiefly standardized to catechins or polyphenols. The contents of caffeine in all extracts (Figure 3) as well as theaflavins in black tea extracts (Figure 4) were also quantified and analogously depicted. As for the outcome of the initial compositional profiling, all commercial samples showed results equivalent to the supplier specification.

**Figure 5:**
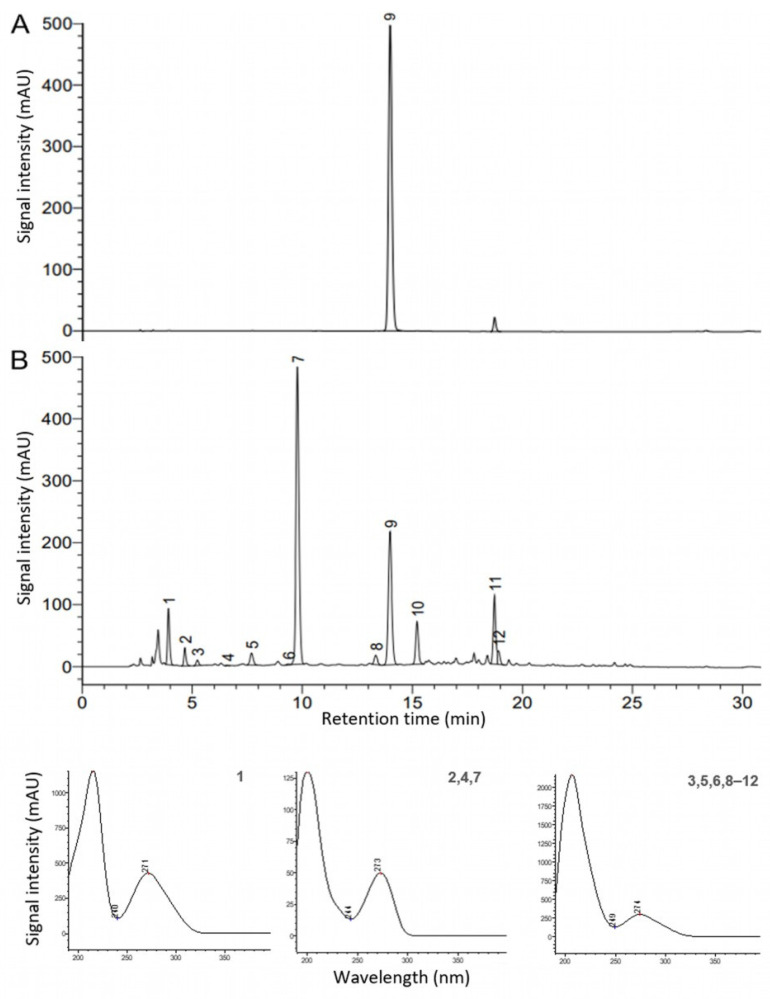
HPLC–DAD chromatograms at 275 nm after the 23-month storage of the (**A**) commercial green tea extract C14 (standardized to 90% EGCG) and (**B**) ADM^®^ Green Tea Extract, showing gallic acid (1), theobromine (2), GC (3), theophylline (4), EGC (5), C (6), caffeine (7), EC (8), EGCG (9), GCG (10), ECG (11), and CG (12), along with UV spectra (190–400 nm) of the bioactive compounds.

**Figure 6:**
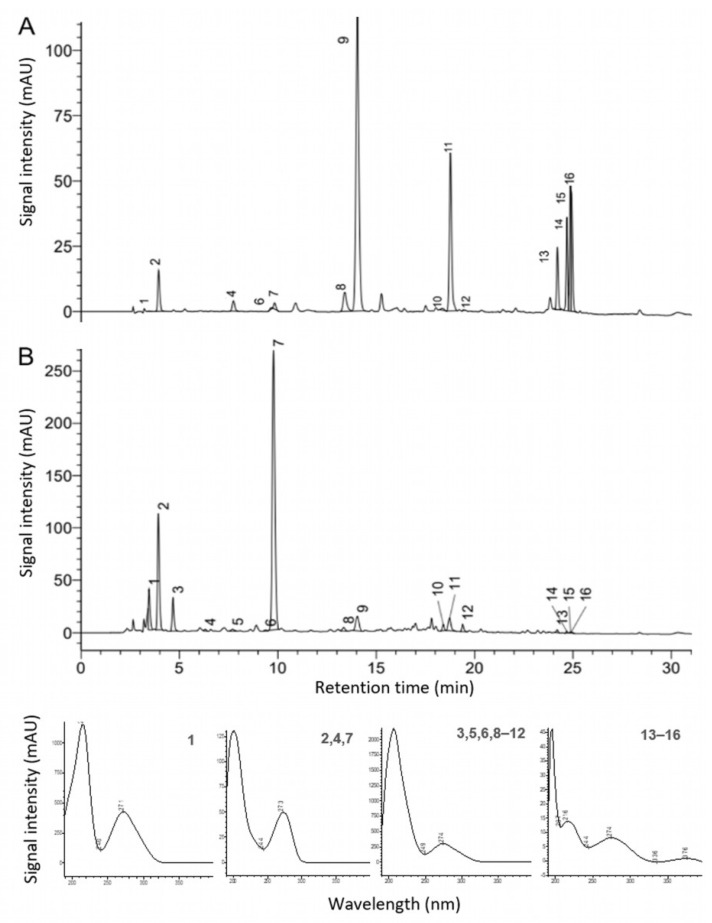
HPLC–DAD chromatograms at 275 nm after the 23-month storage of the (**A**) commercial black tea extract C24 (standardized to 90% EGCG) and (**B**) ADM^®^ Black Tea Extract, showing gallic acid (1), theobromine (2), GC (3), theophylline (4), EGC (5), C (6), caffeine (7), EC (8), EGCG (9), GCG (10), ECG (11), CG (12), and theaflavins (13–16), along with UV spectra (190–400 nm) of the bioactive compounds.

**Figure 7:**
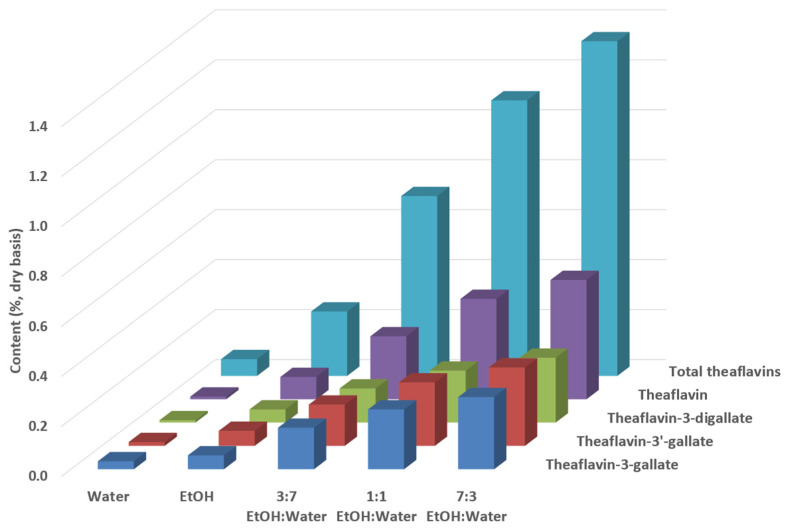
Theaflavin content (%, dry basis) of black tea leaves extracted with water, ethanol (EtOH), and their different mixtures, analyzed via HPLC–DAD.

**Figure 8:**
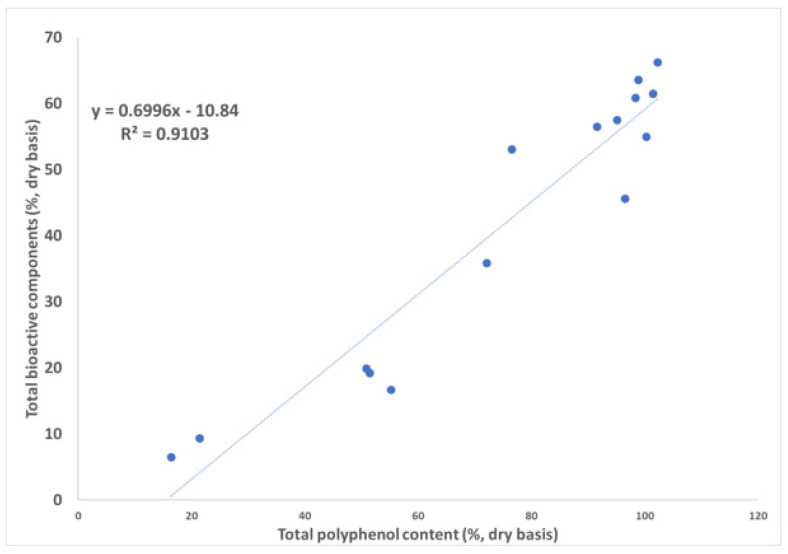
Two-dimensional correlation plot of total polyphenol content (UV method) versus the content of total bioactive components of 15 commercial green tea extracts (HPLC−DAD method).

**Figure 9:**
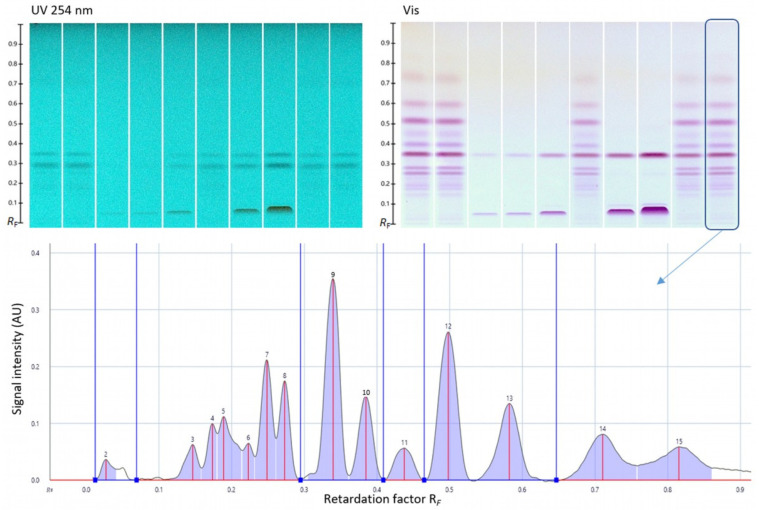
HPTLC chromatograms at UV 254 nm for the detection of methylxanthines and theaflavins, and for the detection of flavan-3-ols, at white light illumination (Vis) after derivatization with the Fast Blue B salt reagent of five batches of the ADM^®^ Tea Complex sample (2 µL/band each, 2.5 mg/mL) along with five levels of the standard mixture consisting of EGCG, theaflavin, and caffeine (S1–S5: 1, 1.5, 4.5, 10.5, and 22.5 µL/band, 37 µg/mL) on the HPTLC plate silica gel 60 RP18 W F_254s_, using a mixture of 1.2 mL acetonitrile, 4 mL water, and 15 mg citric acid, as well as exemplarily for the last sample track, the respective densitogram at 546 nm (absorbance measurement, start zone peak 1 excluded, baseline points as blue lines).

However, we hypothesized that the oxidative stability of the antioxidative molecules may differ throughout the entire shelf life depending on the individual chemical structure and overall composition of the extract. Five commercial tea extracts seemed to be highly purified samples, with a significant concentration of EGCG, the less stable catechin compared to the others such as EGC, ECG, or EC. In fact, the oxidative stability of catechins (EGCG > EGC > ECG > EC) was reported in agreement with the order of the ability for radical formation, which was highest for EGCG [16]. The oxidative stability during the entire shelf life of such highly purified/concentrated tea extract products was questioned because of the absence of other natural antioxidants characteristic for *Camellia sinensis* L., which possibly stabilize the complex mixture. Hence, important bioactive compounds in the powdered tea extracts of different standardizations were again determined close to the end of the product shelf life (Figure 1, Figure 2, Figure 3 and Figure 4, Table 2 and Table 3). As proof, four non-purified/non-concentrated powdered tea extracts were self-produced, *i.e*., ADM^®^ Green/White/Black Tea Extract and Tea Complex. The self-produced extracts were studied over the entire 24-month shelf life (Figure 5 and Figure 6, Table 4). For the optimal self-production of black tea extracts, the extractability of theaflavins was studied with five different solvent systems (Figure 7). Further, it was calculated how the content of individual antioxidative components from tea extracts determined via HPLC–DAD correlated with the total polyphenol content determined via UV spectrometry (Table 5, Figure 8). For the characterization of unknown phenolic compound signals detected in the self-produced extracts, HPLC–ESI–QTOF-MS/MS was used for their tentative assignment (Table 6). Finally, the contents of bioactive compounds in various batches of the self-produced, standardized, and stable Tea Complex sample were quantified via HPLC–DAD versus HPTLC–UV/Vis for method comparison, and thus the results were verified via a second independent chromatographic method (Table 7, Figure 9).

### 3.2. Stability of Bioactive Compounds in 26 Commercial Tea Extracts via HPLC–DAD

Most of the commercial green tea extracts showed a reduction in their EGCG content after 23 months of storage (Figure 1). In green tea extract C14 (standardized to EGCG ≥ 90%), the EGCG content was reduced by about 9% (from 91% to 83%) after 23 months of storage time. The EGCG content of samples C7 (standardized to EGCG ≥ 90%), C13 (standardized to EGCG ≥ 45%), and C18 (standardized to catechins ≥ 15%) was reduced by about 13%, 14%, and 11%, respectively, whereas EGCG in sample C6 (standardized to catechins ≥ 50%) was decreased most by 53% (from 37% to 17%). Some samples such as C2, C15, and C16, whose EGCG content was lower compared to the other samples, showed the same EGCG content after 23 months.

The HPLC–DAD quantification of individual bioactive compounds in the different commercial green tea extracts after 23 months of storage time (Table 2) showed that except sample C2, which was the least purified green tea extract regarding EGCG content (15% EGCG, Table 1), all green tea samples standardized to a higher EGCG content did not comply with the specifications over the claimed product shelf life.

**Table 2:** Individual composition of bioactive components (% dry basis, *n* = 2) from different commercial green tea extracts (C1–C22) analyzed via HPLC–DAD after 23 months of storage time.

Bioactive Compounds *	GA	TB	GC	TP	EGC	C	Caf	EC	EGCG	GCG	ECG	CG	Total Flavan-3-ols	Total Xanthines	Total Bioactives
RT (min)	3.9	4.7	5.3	6.6	7.7	9.7	9.8	13.4	14.0	18.4	18.8	19.0			
C1	0.3 ± 0.0	0.1 ± 0.0	1.0 ± 0.2	0.0 ± 0.0	1.8 ± 0.2	0.2 ± 0.2	0.3 ± 0.0	2.8 ± 0.2	36.8 ± 0.7	6.1 ± 0.2	9.3 ± 0.3	1.1 ± 0.1	59.2 ± 2.4	0.4 ± 0.0	59.8 ± 2.4
C2	2.2 ± 0.0	0.3 ± 0.0	1.3 ± 0.2	0.1 ± 0.0	3.9 ± 0.3	0.0 ± 0.0	2.3 ± 0.0	3.7 ± 0.2	16.4 ± 0.2	2.4 ± 0.2	2.7 ± 0.2	0.5 ± 0.2	30.9 ± 1.5	2.7 ± 0.0	35.8 ± 1.5
C3	0.5 ± 0.1	0.1 ± 0.0	0.4 ± 0.0	0.0 ± 0.0	0.5 ± 0.0	0.3 ± 0.0	2.6 ± 0.6	0.4 ± 0.0	3.0 ± 0.4	0.9 ± 0.2	1.3 ± 0.2	0.4 ± 0.0	7.2 ± 1.2	2.7 ± 0.6	10.3 ± 1.9
C4	0.3 ± 0.0	0.1 ± 0.0	0.4 ± 0.1	0.0 ± 0.0	0.4 ± 0.1	0.4 ± 0.1	0.1 ± 0.0	0.4 ± 0.1	2.7 ± 0.1	0.7 ± 0.1	1.5 ± 0.1	0.4 ± 0.1	6.8 ± 0.8	0.1 ± 0.0	7.2 ± 0.8
C5	0.3 ± 0.0	0.7 ± 0.0	0.5 ± 0.0	0.0 ± 0.0	1.9 ± 0.0	0.3 ± 0.1	2.3 ± 0.0	1.5 ± 0.0	5.2 ± 0.1	0.8 ± 0.0	1.6 ± 0.0	0.3 ± 0.1	12.1 ± 0.2	3.0 ± 0.0	15.4 ± 0.2
C6	2.1 ± 0.0	0.3 ± 0.0	1.4 ± 0.2	0.1 ± 0.0	3.4 ± 0.3	0.8 ± 0.2	2.2 ± 0.0	3.3 ± 0.3	17.0 ± 0.5	4.3 ± 0.3	2.8 ± 0.3	0.7 ± 0.2	33.6 ± 2.1	2.5 ± 0.0	38.2 ± 2.1
C7	0.1 ± 0.0	0.0 ± 0.0	0.5 ± 0.1	0.0 ± 0.0	0.6 ± 0.1	0.0 ± 0.0	0.0 ± 0.0	0.7 ± 0.1	79.2 ± 0.4	0.2 ± 0.2	1.7 ± 0.1	0.0 ± 0.0	82.8 ± 0.6	0.0 ± 0.0	82.9 ± 0.6
C8	0.7 ± 0.0	0.1 ± 0.0	0.6 ± 0.2	0.0 ± 0.0	2.2 ± 0.6	0.7 ± 0.1	0.3 ± 0.0	2.9 ± 0.1	38.5 ± 1.7	2.1 ± 0.2	11.6 ± 0.0	0.7 ± 0.1	59.3 ± 3.1	0.4 ± 0.0	60.4 ± 3.2
C9	0.9 ± 0.0	0.1 ± 0.0	0.7 ± 0.1	0.0 ± 0.0	2.3 ± 0.1	0.6 ± 0.1	0.6 ± 0.1	2.6 ± 0.1	39.3 ± 0.2	2.9 ± 0.1	10.9 ± 0.1	0.8 ± 0.1	60.2 ± 0.7	0.8 ± 0.0	61.9 ± 0.7
C13	0.3 ± 0.0	0.0 ± 0.0	1.0 ± 0.1	0.0 ± 0.0	1.7 ± 0.1	0.0 ± 0.0	0.4 ± 0.0	3.8 ± 0.1	39.8 ± 0.3	5.9 ± 0.1	4.1 ± 0.2	0.7 ± 0.1	55.2 ± 3.2	0.5 ± 0.1	56.0 ± 3.2
C14	0.1 ± 0.0	-	1.6 ± 0.0	-	1.6 ± 0.0	-	-	-	83.3 ± 0.5	1.6 ± 0.0	4.3 ± 0.0	-	92.4 ± 0.2	0.0 ± 0.0	92.5 ± 0.2
C15	0.3 ± 0.0	0.2 ± 0.0	0.5 ± 0.1	0.0 ± 0.0	0.7 ± 0.1	0.0 ± 0.0	0.1 ± 0.0	0.5 ± 0.1	1.7 ± 0.1	0.7 ± 0.1	0.7 ± 0.1	0.4 ± 0.1	5.2 ± 2.1	0.2 ± 0.0	5.8 ± 2.1
C16	0.1 ± 0.0	0.5 ± 0.0	0.7 ± 0.2	0.0 ± 0.0	3.8 ± 0.1	0.6 ± 0.2	4.0 ± 0.0	1.3 ± 0.2	5.4 ± 0.2	0.4 ± 0.2	1.0 ± 0.2	0.5 ± 0.2	14.4 ± 1.2	4.6 ± 0.0	19.2 ± 1.2
C17	0.6 ± 0.0	0.1 ± 0.0	0.7 ± 0.1	0.0 ± 0.0	3.2 ± 0.1	0.7 ± 0.1	0.6 ± 0.0	2.8 ± 0.1	37.1 ± 0.1	1.7 ± 0.0	8.1 ± 0.0	0.6 ± 0.1	54.9 ± 0.6	0.7 ± 0.0	56.2 ± 0.7
C18	0.1 ± 0.0	0.3 ± 0.0	0.8 ± 0.2	0.0 ± 0.0	1.2 ± 0.2	0.5 ± 0.2	6.7 ± 0.0	0.5 ± 0.1	6.3 ± 0.2	2.1 ± 0.2	1.2 ± 0.2	0.6 ± 0.2	13.3 ± 0.5	7.0 ± 0.0	20.3 ± 0.5
C19	3.5 ± 0.0	0.2 ± 0.0	1.1 ± 0.1	0.0 ± 0.0	3.2 ± 0.2	0.8 ± 0.2	3.0 ± 0.0	3.5 ± 0.1	18.8 ± 0.0	2.3 ± 0.2	8.4 ± 0.1	0.8 ± 0.2	38.9 ± 0.9	3.2 ± 0.0	45.6 ± 0.9
C20	0.8 ± 0.0	0.0 ± 0.0	0.7 ± 0.1	0.0 ± 0.0	2.4 ± 0.1	0.8 ± 0.1	0.2 ± 0.0	3.0 ± 0.1	45.1 ± 0.5	3.6 ± 0.1	6.4 ± 0.1	0.6 ± 0.1	62.5 ± 1.3	0.3 ± 0.0	63.5 ± 1.3
C21	0.2 ± 0.0	0.1 ± 0.0	0.4 ± 0.1	0.0 ± 0.0	0.5 ± 0.1	0.4 ± 0.1	0.1 ± 0.0	0.4 ± 0.1	0.6 ± 0.1	0.5 ± 0.1	0.5 ± 0.1	0.4 ± 0.1	3.6 ± 0.0	0.2 ± 0.0	4.0 ± 0.0
C22	0.1 ± 0.0	0.1 ± 0.0	0.0 ± 0.0	0.2 ± 0.2	0.0 ± 0.0	0.0 ± 0.0	0.0 ± 0.0	0.0 ± 0.0	0.4 ± 0.0	0.3 ± 0.0	0.0 ± 0.0	0.0 ± 0.0	0.7 ± 0.1	0.2 ± 0.0	1.0 ± 0.1

* Gallic acid (GA), theobromine (TB), (+)-gallocatechin (GC), theophylline (TP), (−)-epigallocatechin (EGC), (+)-catechin (C), caffeine (Caf), (−)-epicatechin (EC), epigallocatechin gallate (EGCG), (−)-gallocatechin gallate (GCG), epicatechin gallate (ECG), (−)-catechin 3-gallate (CG).

In fact, EGCG in sample C2 corresponded to less than 50% of total catechins plus methylxanthines (47%, Table 2), whereas in samples C7 and C14, with a higher content of total flavan-3-ols, the EGCG content conformed to about 96% (83.3% versus 92.4%) and 90% (79.2% versus 82.8%; Table 2), respectively, of the total flavan-3-ols and methylxanthines (the latter was zero). Standardized to L-theanine, samples C21 (3.6%, dry basis) and C22 (0.7%, dry basis) showed a comparatively lower flavan-3-ol content. Most of the green tea extracts were decaffeinated, and only samples C16 and C18 showed a substantial caffeine content (4.6% and 7.0%, respectively, dry basis).

Some of the seven commercial black tea extracts (Table 3, Figure 2) also showed a decrease in the EGCG content after 23 months of storage, *i.e*., the EGCG content of sample C11 decreased by 13% (from 40% to 35%), that of C12 by 23% (from 44% to 34%), and that of C24 by 9% (from 29% to 27%). This observed instability during the product shelf life was explained by the high EGCG content of samples C11 and C12, which was over 50% of total catechins (64% and 68%, respectively, Table 3). Most of the commercial black tea extracts seemed to be previously decaffeinated, except C25 (4.1% caffeine, Table 3).

**Table 3:** Content of individual bioactive components (%, dry basis, *n* = 2) in seven commercial black tea extracts (C10–C26) analyzed via HPLC–DAD at the end of the product shelf life.

Bioactive Components *	RT	C10	C11	C12	C23	C24	C25	C26
GA	3.9	7.8 ± 2.0	1.0 ± 0.0	1.2 ± 0.0	0.1 ± 0.0	1.2 ± 0.0	0.4 ± 0.0	0.5 ± 0.0
TB	4.7	0.1 ± 0.0	0.1 ± 0.0	0.1 ± 0.0	0.0 ± 0.0	0.0 ± 0.0	0.7 ± 0.0	0.1 ± 0.0
GC	5.3	3.1 ± 0.9	0.7 ± 0.0	0.9 ± 0.2	0.0 ± 0.0	0.7 ± 0.2	0.9 ± 0.0	0.7 ± 0.1
TP	6.6	0.1 ± 0.0	0.0 ± 0.0	0.0 ± 0.0	0.0 ± 0.0	0.0 ± 0.0	0.0 ± 0.0	0.0 ± 0.0
EGC	7.7	3.4 ± 0.9	2.4 ± 0.0	3.1 ± 0.2	0.0 ± 0.0	1.4 ± 0.2	1.8 ± 0.0	0.7 ± 0.1
C	9.7	3.3 ± 0.9	0.7 ± 0.0	0.8 ± 0.2	0.0 ± 0.0	0.8 ± 0.2	0.6 ± 0.0	0.7 ± 0.0
Caf	9.9	0.2 ± 0.1	0.6 ± 0.0	0.5 ± 0.0	2.3 ± 0.0	0.4 ± 0.0	4.1 ± 0.0	2.6 ± 0.0
EC	13.4	3.7 ± 0.9	2.7 ± 0.0	0.6 ± 0.2	0.6 ± 0.1	2.5 ± 0.2	1.3 ± 0.1	0.7 ± 0.0
EGCG	14.0	7.6 ± 0.9	34.8 ± 0.1	33.8 ± 0.4	0.7 ± 0.1	26.5 ± 0.2	4.7 ± 0.0	1.0 ± 0.1
GCG	18.4	3.5 ± 0.9	2.5 ± 0.1	2.3 ± 0.2	0.6 ± 0.1	1.7 ± 0.2	1.3 ± 0.0	0.9 ± 0.0
ECG	18.8	4.8 ± 0.9	10.4 ± 0.0	7.5 ± 0.2	2.3 ± 0.0	11.0 ± 0.2	1.2 ± 0.5	0.8 ± 0.0
CG	19.0	3.3 ± 0.9	0.7 ± 0.1	0.7 ± 0.2	0.8 ± 0.1	0.9 ± 0.2	1.3 ± 0.8	0.7 ± 0.0
Theaflavin	24.2	3.9 ± 0.1	0.0 ± 0.0	0.0 ± 0.0	7.9 ± 0.9	2.2 ± 0.0	0.0 ± 0.0	0.0 ± 0.0
Theaflavin-3′-gallate	24.7	6.4 ± 0.0	0.0 ± 0.0	0.0 ± 0.0	14.7 ± 0.3	3.4 ± 0.0	0.0 ± 0.0	0.0 ± 0.0
Theaflavin-3,3′-digallate	24.8	7.7 ± 0.2	0.0 ± 0.0	0.0 ± 0.0	21.6 ± 0.4	2.9 ± 0.1	0.0 ± 0.0	0.0 ± 0.0
Theaflavin-3-gallate	24.9	3.5 ± 0.0	0.0 ± 0.0	0.0 ± 0.0	9.1 ± 0.4	2.4 ± 0.6	0.0 ± 0.0	0.0 ± 0.0
Total flavan-3-ols		32.8 ± 8.1	54.0 ± 0.2	49.7 ± 2.1	4.9 ± 1.2	45.5 ± 0.5	13.2 ± 1.3	6.1 ± 0.0
Total methylxanthines		0.3 ± 0.1	0.7 ± 0.0	0.6 ± 0.0	2.3 ± 0.0	0.4 ± 0.0	4.8 ± 0.0	2.6 ± 0.2
Total theaflavins		21.5 ± 0.4	0.0 ± 0.0	0.0 ± 0.0	53.3 ± 0.0	10.9 ± 0.0	0.0 ± 0.0	0.0 ± 0.0
Total bioactive components		62.4 ± 6.4	56.6 ± 0.2	51.5 ± 2.1	60.5 ± 1.2	58.1 ± 0.5	18.4 ± 1.3	9.3 ± 0.0

* Gallic acid (GA), theobromine (TB), (+)-gallocatechin (GC), theophylline (TP), (−)-epigallocatechin (EGC), (+)-catechin (C), caffeine (Caf), (−)-epicatechin (EC), epigallocatechin gallate (EGCG), (−)-gallocatechin gallate (GCG), epicatechin gallate (ECG), (−)-catechin 3-gallate (CG).

The caffeine content of the commercial tea extracts was analogously studied initially and just before the end of product shelf life (Figure 3). Some green tea samples seem to keep their caffeine content during the entire shelf life (C2, C15, C16, and C17); however, other samples (C6, C8, C9, and C20) showed a reduction of about 20–40% in the caffeine content after storage. Although difficult to spot due to the low content, the caffeine content of C6 was reduced from 4.2% to 2.2%, that of C8 from 0.5% to 0.3%, that of C9 from 0.8% to 0.6%, and that of C20 from 0.4% to 0.2%.

Some samples lost their initial caffeine content almost completely after the claimed product shelf life, such as C1 whose caffeine content decreased by 80% (from 1.6% to 0.3%). Most likely, the previously observed decrease in the catechin content affected not only the overall stability of the product but also other compounds such as caffeine. Apart from that, caffeine and individual catechins showed affinity by repulsive and attractive intermolecular forces [17], which may explain the impact on the stability of caffeine during the product shelf life.

Finally, the content of theaflavins in the seven black tea extracts was also affected during the product shelf life, but to a lesser extent (Figure 4). Only sample C10 showed a specified content of theaflavins (40%, dry basis) different from that obtained in our laboratory in the initial analysis (25%, dry basis). The reduction after 23 months of storage was 13% (from 25% to 22%, dry basis). A similar decrease of 12% and 8% was observed for samples C23 and C24, respectively. The decrease in the content of theaflavins during the product shelf life of some commercial samples could be explained by the vulnerability of this oligomer to oxidation. In fact, some monomeric flavan-3-ols can be even more stable than theaflavins [18,19]. However, more black tea samples standardized to theaflavins must be studied to complement these first results.

### 3.3. Stability of Bioactive Compounds in Self-Produced Tea Extracts via HPLC–DAD

Especially the green and black tea extracts standardized to a higher EGCG content showed a decrease in their bioactive compounds over the claimed product shelf life. Hence, four different non-purified extracts were self-produced and analyzed analogously. The HPLC–DAD chromatogram at 275 nm after the 23-month storage of the commercial green tea sample C14 (standardized to 90% EGCG) was compared with that of the self-produced non-purified ADM^®^ Green Tea Extract (standardized to 40–60% polyphenols). Both extracts were obtained via water extraction. As expected, the highly purified extract C14 showed almost only the EGCG (Figure 5A), which was reduced by 8% over the product shelf life. In contrast, the ADM^®^ Green Tea Extract showed gallic acid, methylxanthines, and flavan-3-ols (Figure 5B), and its EGCG content remained over 8% (dry basis) throughout its product shelf life according to the specifications. In fact, its EGCG represented less than 50% of total catechins (over 20%, dry basis), which underlines our hypothesis that the oxidative stability of EGCG throughout the product shelf life can be stabilized by other compounds present. Thus, the overall composition of the extract has a substantial impact on stability during the shelf life of the product.

The HPLC–DAD chromatogram at 275 nm of the 23-month-stored commercial black tea extract C24 (standardized to 10% theaflavins, Figure 6A) was compared with that of the self-produced non-purified ADM^®^ Black Tea Extract (standardized to 10% of polyphenols, Figure 6B). Both extracts were obtained via water extraction. The caffeine content in sample C24 was 10-fold lower compared to the ADM^®^ Black Tea Extract (0.4% and 4.0%, respectively, dry basis). However, the EGCG and theaflavin content was much higher in C24 than in ADM^®^ Black Tea extract with a total theaflavins content of 0.1%, dry basis.

Theaflavins were only slightly extracted from black tea leaves with water or ethanol as individual solvents, whereas an adequate mixture of ethanol and water improved their extraction (Figure 7, analyzed via HPLC). The extractant mixture of ethanol and water 70:30, *v*/*v*, resulted in the highest extractability of theaflavins since it showed a 21-fold and 5-fold higher content of total theaflavins compared to water (0.07 versus 1.34%, dry basis) and ethanol (0.26 versus 1.34%, dry basis), respectively.

Two further self-produced non-purified tea extracts, *i.e*., ADM^®^ Tea Complex and ADM^®^ White Tea Extract, were analyzed. For both extracts, the standardized EGCG content was lower than 50% of total flavan-3-ols (48% and 45%, respectively). At different time-points during their shelf life (after a storage of 0, 6, 12, and 24 months), the content of the individual bioactive compounds did not change (Table 4). It is clear that a balanced composition with a complete profile of flavan-3-ols, methylxanthines, theaflavins (for black teas), and phenolic acids such as gallic acid preserved them from oxidation. The ADM^®^ Tea Complex showed only slight variations during the entire product shelf life with flavan-3-ols in the range 11.6–12.3%, methylxanthines in the range 6.4–7.7% and theaflavins of 0.1%, all referring to dry basis. For the ADM^®^ White Tea Extract, flavan-3-ols were in the range 10.3–10.8% and methylxanthines in the range 5.4–6.5% during the entire product shelf life, all referring to dry basis. Thus, these products were in agreement with the claimed specification (Table 1) during the entire product shelf life.

**Table 4:** Group-specific composition (% dry basis, *n* = 2) of the self-produced non-purified ADM^®^ Tea Complex and White Tea Extract analyzed at different storage months via HPLC–DAD.

ADM^®^ Extract Tea Complex	Catechins as EGCG * eq. (% Dry Basis)	Xanthines as Caffeine eq. (% Dry Basis)	Theaflavins (% Dry Basis)
Control	11.6 ± 0.0	6.4 ± 0.2	0.1 ± 0.0
6 Months	13.0 ± 0.0	6.6 ± 0.4	0.1 ± 0.0
12 Months	11.7 ± 0.2	7.7 ± 0.1	0.1 ± 0.0
24 Months	12.3 ± 1.6	6.5 ± 0.0	0.1 ± 0.0
**White Tea**			
Control	10.8 ± 0.8	6.1 ± 0.2	-
6 Months	10.3 ± 0.2	5.4 ± 0.0	-
12 Months	10.3 ± 1.2	6.5 ± 0.5	-
24 Months	10.3 ± 0.1	5.8 ± 0.1	-

* (−)-epigallocatechin gallate (EGCG).

In addition, tea samples enriched with EGCG may cause toxicity even at lower dosages as reported by Commission Regulation (EU) 2022/2340 [12]. Therefore, an objective standardization considering a moderate concentration of important bioactive components is preferable to protect the product from oxidation and also to comply with safety regulations.

As it happens with tea leaves or ready-to-drink tea beverages, the tea powdered extracts are susceptible to oxidation, but to a lesser extent for those extracts not highly purified and with a more complete profile of characteristic antioxidants from tea. Apart from that, the container or packaging is crucial to preserve these molecules from light, humidity, and/or oxygen throughout the shelf life [13,14].

### 3.4. Correlation to the Total Polyphenol Content

The total polyphenol content was reported to correlate linearly with different antioxidant capacity measurement methods such as DPPH• assay, ORAC assay, ABTS assay, *etc*. [20]. However, it was of interest to know how individual bioactive compounds or compound groups, such as flavan-3-ols and methylxanthines, may correlate with the total polyphenol content. The latter was taken from the specifications of the respective commercial tea extracts and correlated with the content of the individual bioactive compounds in 15 green tea extracts obtained via HPLC–DAD. Individual methylxanthines showed low correlation with the total polyphenol content (caffeine R = 0.43, theophylline R = 0.02, and theobromine R = 0.41, Table 5). The same low correlation was observed for total methylxanthines (R = 0.41). On the contrary, higher individual correlations with the total polyphenol content were observed for flavan-3-ols (EGCG R = 0.87 and ECG R = 0.88). Total flavan-3-ols showed the best correlation (R = 0.93) for this molecule group.

The highest correlation (R = 0.95, Figure 8) was obtained for total phenol content according to Folin–Ciocalteu (UV method) versus the content of total bioactive components (HPLC–DAD method). Hence, the observed decrease in EGCG not only affects the standardization of the product, but also other quality marker such as total polyphenol content because of the linear correlation with this component [3]. The best correlation for total bioactive components suggests that a more comprehensive tea profile with the presence of key bioactive components protects the product from oxidation during its shelf life.

**Table 5:** Linear correlations of total polyphenol content (UV method) with contents of individual bioactive compounds, compound groups, and total bioactive components (HPLC–DAD method) of 15 commercial green tea extracts (as in Figure 8).

Bioactive Components *	Slope a x	Intercept b	Determination Coefficient R^2^	Correlation Coefficient R
GA	0.56	0.01	0.03	0.16
TB	0.00	0.46	0.17	0.41
GC	0.00	0.67	0.07	0.27
TP	0.00	0.00	0.00	0.02
EGC	0.02	1.08	0.30	0.54
C	0.00	0.50	0.02	0.15
Caf	−0.02	3.03	0.18	0.43
EC	0.03	0.17	0.48	0.69
EGCG	0.49	−15.37	0.76	0.87
GCG	0.03	−0.22	0.46	0.68
ECG	0.12	−3.64	0.78	0.88
CG	0.00	0.59	0.04	0.20
Total catechins	0.71	−16.06	0.87	0.93
Total methylxanthines	86.28	−6.43	0.17	0.41
Total bioactive components	0.07	−10.84	0.91	0.95

* Gallic acid (GA), theobromine (TB), (+)-gallocatechin (GC), theophylline (TP), (−)-epigallocatechin (EGC), (+)-catechin (C), caffeine (Caf), (−)-epicatechin (EC), epigallocatechin gallate (EGCG), (−)-gallocatechin gallate (GCG), epicatechin gallate (ECG), (−)-catechin 3-gallate (CG).

### 3.5. Characterization of Unidentified Components via HPLC–ESI–QTOF-MS/MS

Unidentified compound signals in the HPLC–DAD chromatograms of the self-produced non-purified tea extracts were further characterized via HPLC–ESI–QTOF-MS/MS analysis in the negative ionization mode. It complemented, though preliminarily assigned, the list of phenolic compounds (Table 6). All caffeoylquinic acid isomers eluted at 12.5 min and showed the deprotonated molecule [M − H]^−^ at *m*/*z* 353.0878, together with the fragments at *m*/*z* 179/191/195 for 4-caffeoylquinic acid, at *m*/*z* 169/191/151/173/187 for 5-caffeoylquinic acid, and at *m*/*z* 191/291 for chlorogenic acid. Both enantiomers of gallocatechin, *i.e.*, (+)-GC and (−)-GC, eluted at 13.9 min and showed the deprotonated molecule [M − H]^−^ at *m*/*z* 305.0676.

**Table 6:** List of phenolic compounds in the self-produced non-purified tea extracts, preliminary assigned via HPLC–ESI–QTOF-MS.

Compound Name	Score	Formula	Intensity	Expected *m*/*z*	Found *m*/*z*	Δ (ppm)	Fragment Observed *m*/*z*	RT (min)
1-Caffeoylquinic acid	86%	C16H18O9	9130	353.0878	353.0878	3.0	-	12.5
4-Caffeoylquinic acid	86%	C16H18O9	9130	353.0878	353.0878	3.0	179/191/195	12.5
5-Caffeoylquinic acid	86%	C16H18O9	9130	353.0878	353.0878	3.0	169/191/151/173/187	12.5
Chlorogenic acid	86%	C16H18O9	9130	353.0878	353.0878	3.0	191/291	12.5
(+)-Gallocatechin	61%	C15H14O7	11,650	305.0667	305.0676	0.3	-	13.9
(−)-Gallocatechin	61%	C15H14O7	11,650	305.0667	305.0676	0.3	-	13.9
Galloyl glucose	71%	C13H16O10	25,124	331.0671	331.0685	0.3	169	8.1
Galloylquinic acid	74%	C14H16O10	111,763	343.0671	343.0687	0.9	-	5.6

### 3.6. Quantitative Verification via HPTLC–UV/Vis

In a previous study [11], all samples were already analyzed for their characteristic effect-directed profiles via HPTLC bioprofiling. HPTLC was combined with enzymatic or biological planar assays to answer important questions regarding the bioactivity of the functional extracts. Such hyphenated HPTLC is a highly straightforward tool to find effects in complex samples [21,22,23,24,25]. Here, as an alternative method, HPTLC–UV/Vis was used for comparative quantification and for the verification of the obtained HPLC–DAD results via a second independent chromatographic method. As an example, the ADM^®^ Tea Complex sample was selected and dissolved in methanol, since dimethyl sulfoxide had to be avoided in HPTLC due to its low vapor pressure. This solvent change was found to be appropriate, since it was made for both the HPLC and the HPTLC method for this comparison. For selective HPTLC detection, a simple derivatization with the Fast Blue B salt reagent was performed, followed by absorbance measurement. This led post-chromatographically to an increase in zone resolution (compensates for the poor peak capacity) because the interested compound class was specifically detected, but no matrix (Figure 9).

The contents of the bioactive compound groups in five batches of the standardized and stable ADM^®^ Tea Complex Extract were quantified via HPTLC–UV/Vis and via HPLC–DAD. The results obtained (Table 7) were compared and found to be highly comparable using two independent orthogonal chromatographic methods.

**Table 7:** Comparison of the determined contents via HPTLC–UV/Vis versus HPLC–DAD of the three bioactive compound groups and total bioactive compounds in the ADM^®^ Tea Complex sample.

Bioactive Compound Content (%)	One Sample		Mean of Five Batches (*n* = 5)	
	HPTLC–UV/Vis	HPLC–DAD	HPTLC–UV/Vis	HPLC–DAD
EGCG * equivalents	12.6	10.9	14.7	11.9
Methylxanthines	4.3	6.5	6.4	6.2
Theaflavin equivalents	0.1	0.1	0.3	0.2
Total bioactive compounds	17.0	17.5	21.3	18.1

* (−)-epigallocatechin gallate (EGCG).

## 4. Conclusions

Liquid chromatographic techniques proved useful to evaluate the quality and stability of tea extracts. Comprehensive compositional profiles were obtained for 30 tea extract samples using HPLC–DAD and HPTLC–UV/Vis. The analyzed contents of important bioactive compounds in the commercial tea extract samples at the end of the product shelf life differed much from what was claimed by the suppliers. Most of the commercial green and black tea extracts showed a decrease in the content of flavan-3-ols, which are the main bioactive components in tea. Focusing on EGCG, the major flavan-3-ol, most of the samples highly concentrated in this molecule showed a decrease at the end of the product shelf life, which compromised the standardization claimed by the supplier. Only the samples with a moderate EGCG content, corresponding to less than 50% of the total flavan-3-ols, maintained the original standardization throughout the shelf life. A high EGCG concentration to the detriment of the other characteristic flavan-3-ols or antioxidant molecules normally present in tea (flavanols, phenolic acids, caffeoylquinic acids, *etc*.) could probably explain the lower oxidative stability of the product, since EGCG is the less stable catechin. The decrease in EGCG not only affected the standardization of the product, but also other quality markers such as the total polyphenol content. A balanced composition with a complete profile of flavan-3-ols, methylxanthines, theaflavins (for black tea), and phenolic acids such as gallic acid preserved from oxidation during the product shelf life and may better comply with safety regulations. Caffeine showed a decrease in most of the commercial green and black tea extracts in line with the decrease of the catechins. The observed decrease in the catechin content affected the overall stability of the product, and most likely, of other compounds such as caffeine. The decrease in the content of theaflavins during the product shelf life of some commercial samples was explained by their vulnerability to oxidation.

## Figures and Tables

**Table 1:** The 26 different commercial powdered green and black tea extracts bought as dietary supplements or nutraceuticals on the market with different standardizations as well as 4 self-produced extracts; all samples were also analyzed via HPTLC bioprofiling elsewhere [11].

19 Green Tea Extracts	Standardization	Analysis
C1	Polyphenols (98%), catechins (85%), EGCG * (50%)	UV, HPLC
C2	Polyphenols (70%), catechins (35%), EGCG (15%)	UV, HPLC
C3	L-Theanine 20%	HPLC
C4	L-Theanine 40%	HPLC
C5	Polyphenols (50%)	UV
C6	Catechins (≥50%)	HPLC
C7	EGCG (≥90%)	HPLC
C8	Polyphenols (98%), catechins (75%), EGCG (45%)	UV, HPLC
C9	Polyphenols (95%), catechins (60%), EGCG (45%)	UV, HPLC
C13	EGCG (≥45%)	HPLC
C14	EGCG (≥90%)	HPLC
C15	Polyphenols (15%)	UV
C16	Polyphenols (50%)	UV
C17	Polyphenols (50%)	UV
C18	Catechins (≥15%)	UV
C19	Polyphenols (90%), catechins (60%), EGCG (40%)	UV, HPLC
C20	Polyphenols (95%), catechins (80%), EGCG (55%)	UV, HPLC
C21	L-Theanine 20%	HPLC
C22	L-Theanine 60%	HPLC
**7 Black Tea Extracts**		
C10	Polyphenols (70%), theaflavins (40%)	HPLC
C11	Polyphenols (95%), catechins (70%), EGCG (40%)	HPLC
C12	Polyphenols (98%), catechins (70%), EGCG (40%)	HPLC
C23	Theaflavins 60%	HPLC
C24	Theaflavins 10%	HPLC
C25	Polyphenols 50%	UV
C26	Polyphenols 20%	UV
**4 Self-Produced ADM^®^ Extracts**		
Green Tea	20% flavan-3-ols, 8% EGCG, 40–60% polyphenols	HPLC
White Tea	7% flavan-3-ols (monomeric), 3% xanthines	HPLC
Black Tea	10% total polyphenols, 3% caffeine	HPLC
Tea Complex	10% flavan-3-ols (monomeric and theaflavins), 5% xanthines	HPLC, HPTLC

* (−)-epigallocatechin gallate (EGCG).

## Data Availability

Data is contained within the article.

## Supplementary Materials

The following supporting information can be downloaded at: https://www.mdpi.com/article/10.3390/antiox12122121/s1, Figure S1: Chromatogram obtained at 275 nm from the green tea extract C21, standardized to L-Theanine 20%. In comparison, other commercial green tea extracts such as C14 (Figure 5) had a much higher content of bioactive compounds, for which the signal intensity had to be scaled to 500 mAU. Figure S2: Chromatogram obtained at 275 nm from the green tea extract C22, standardized to L-Theanine 60%. In comparison, other commercial green tea extracts such as C14 (Figure 5) had a much higher content of bioactive compounds, for which the signal intensity had to be scaled to 500 mAU. Figure S3: Chromatogram obtained at 275 nm from the green tea extract C3, standardized to L-Theanine 40%. In comparison, other commercial green tea extracts such as C14 (Figure 5) had a much higher content of bioactive compounds, for which the signal intensity had to be scaled to 500 mAU. Figure S4: Chromatogram obtained at 275 nm from the green tea extract C4, standardized to L-Theanine 20%. In comparison, other commercial green tea extracts such as C14 (Figure 5) had a much higher content of bioactive compounds, for which the signal intensity had to be scaled to 500 mAU.
