# Supplementary material for: Stability of Flavan-3-ols, Theaflavins, and Methylxanthines in 30 Industrial Green, Black, and White Tea (Camellia sinensis L.) Extracts Characterized via Liquid Chromatography Techniques
Source: Antioxidants (Basel). 2023 Dec 15;12(12):2121. doi: 10.3390/antiox12122121 (PMC10740512; doi:10.3390/antiox12122121)
Supplement: Supplementary file 1 [file antioxidants-12-02121-s001.zip › antioxidants-2738137-supplementary.pdf]

Supplementary Material

Stability of Flavan-3-ols, Theaflavins, and Methylxanthines in 30 Industrial Green, Black, and White Tea (*Camellia sinensis* L.) Extracts Characterized via Liquid Chromatography Techniques

Antonio M. Inarejos-Garcia [1], Julia Heil [2], Sonia Guilera Bermell [1] and Gertrud E. Morlock [2,3,*]

[1] Department of Functional Extracts, ADM® Wild Valencia, 46740 Carcaixent, Spain

[2] Institute of Nutritional Science, Chair of Food Science, and TransMIT Center for Effect-Directed Analysis, Justus Liebig University Giessen, Heinrich-Buff-Ring 26-32, 35392 Giessen, Germany

[3] Center for Sustainable Food Systems, Justus Liebig University Giessen, Senckenbergstr. 3, 35390 Giessen, Germany

[*] Correspondence: gertrud.morlock@uni-giessen.de; Tel.: +49-6419939140

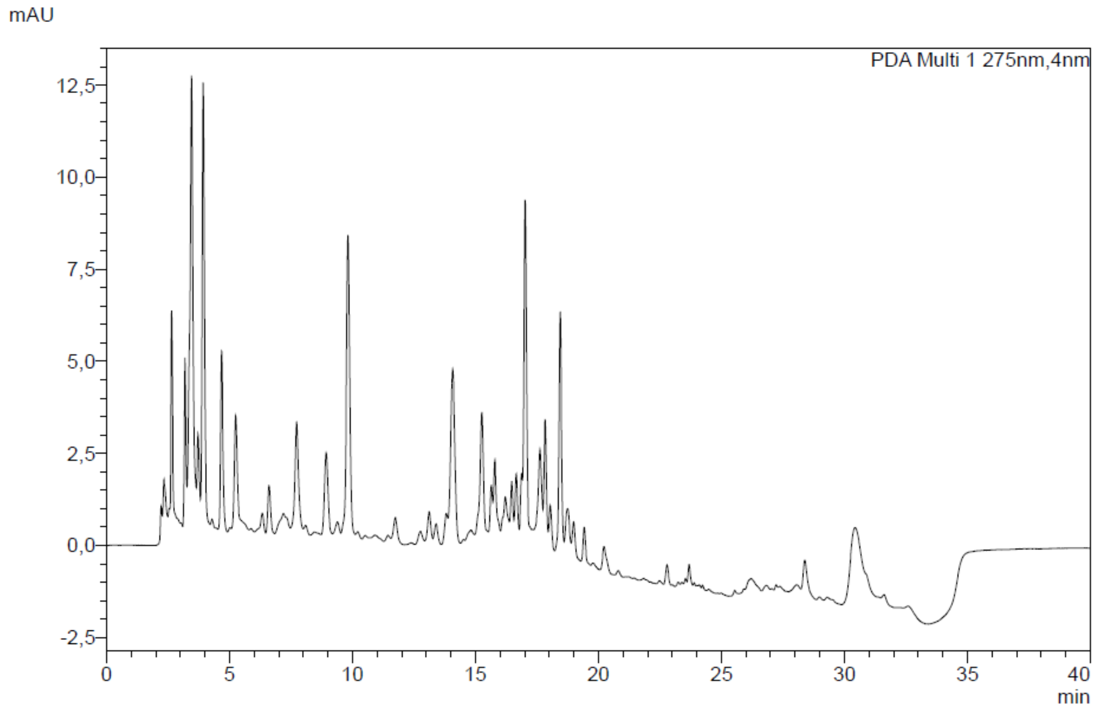

Figure S1. Chromatogram obtained at 275 nm from the green tea extract C21, standardized to L-Theanine 20%. In comparison, other commercial green tea extracts such as C14 (Figure 5) had a much higher content of bioactive compounds, for which the signal intensity had to be scaled to 500 mAU.

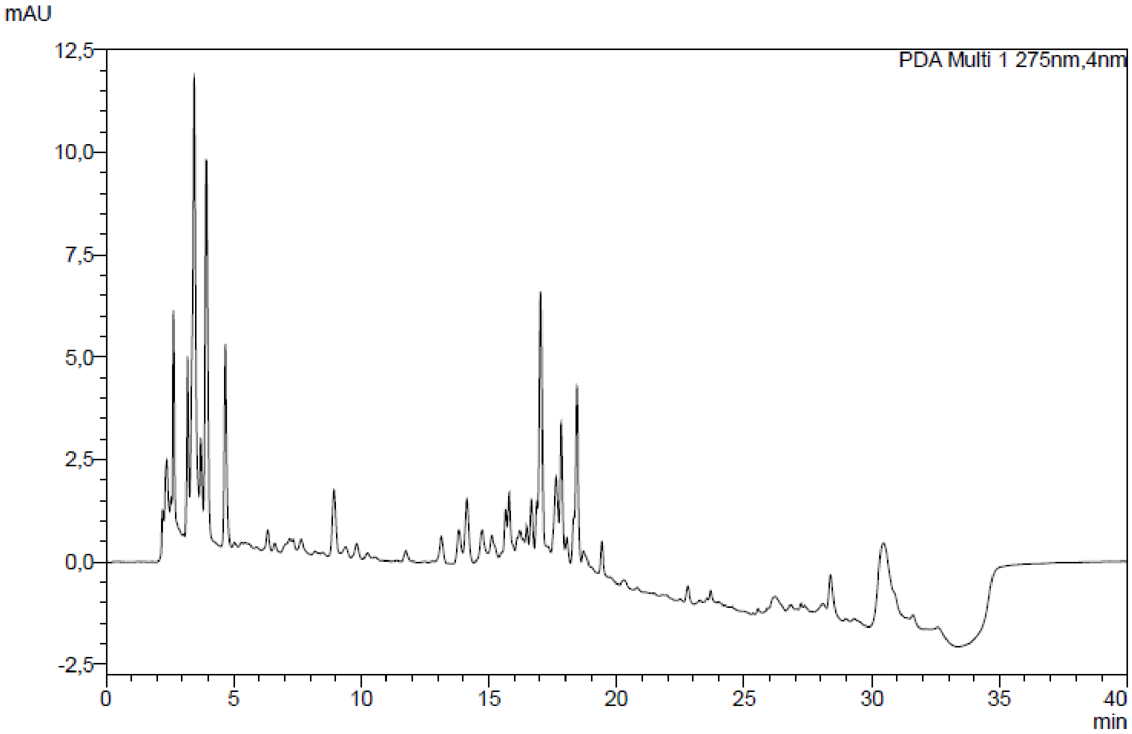

Figure S2. Chromatogram obtained at 275 nm from the green tea extract C22, standardized to L-Theanine 60%. In comparison, other commercial green tea extracts such as C14 (Figure 5) had a much higher content of bioactive compounds, for which the signal intensity had to be scaled to 500 mAU.

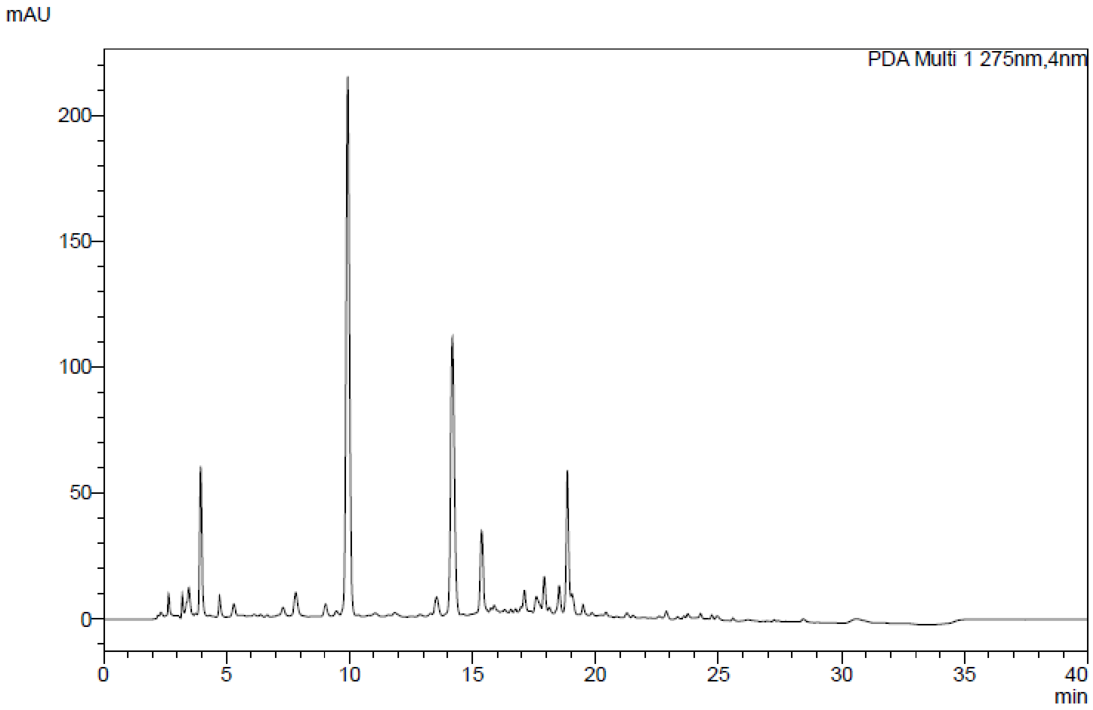

Figure S3. Chromatogram obtained at 275 nm from the green tea extract C3, standardized to L-Theanine 40%. In comparison, other commercial green tea extracts such as C14 (Figure 5) had a much higher content of bioactive compounds, for which the signal intensity had to be scaled to 500 mAU.

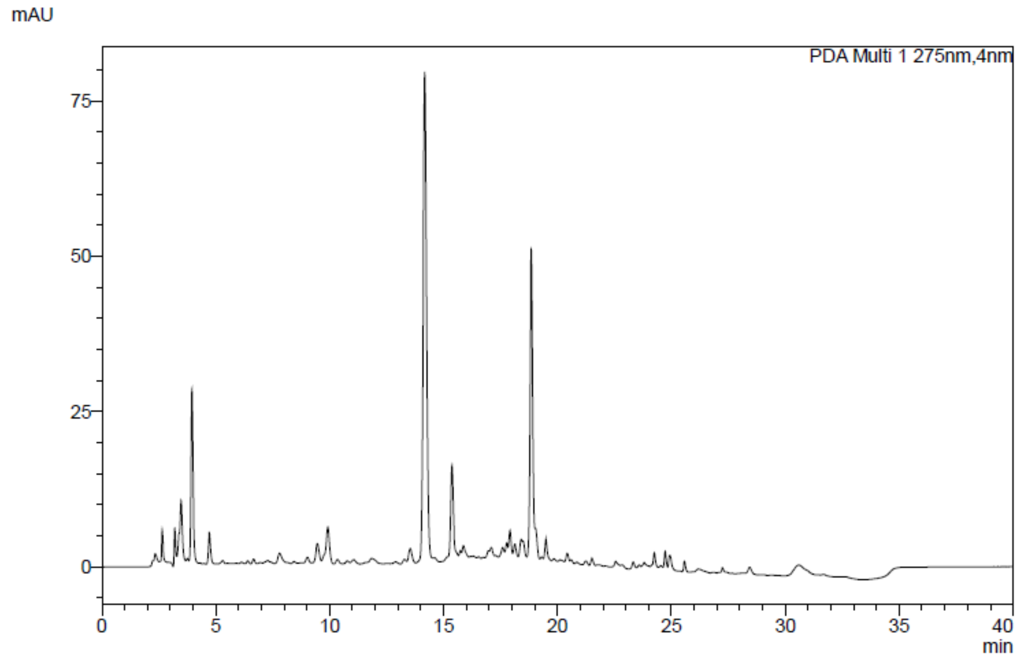

Figure S4. Chromatogram obtained at 275 nm from the green tea extract C4, standardized to L-Theanine 20%. In comparison, other commercial green tea extracts such as C14 (Figure 5) had a much higher content of bioactive compounds, for which the signal intensity had to be scaled to 500 mAU.
